# The Effect of Post-harvest Conditions in *Narcissus* sp. Cut Flowers Scent Profile

**DOI:** 10.3389/fpls.2020.540821

**Published:** 2021-01-07

**Authors:** Marta I. Terry, Victoria Ruiz-Hernández, Diego J. Águila, Julia Weiss, Marcos Egea-Cortines

**Affiliations:** ^1^Genética Molecular, Instituto de Biotecnología Vegetal, Universidad Politécnica de Cartagena, Cartagena, Spain; ^2^Department of Biosciences, University of Salzburg, Salzburg, Austria; ^3^Las Cabezuelas Sociedad Cooperativa, Alhama de Murcia, Spain

**Keywords:** circadian rhythm, constitutive volatiles, floral scent, gcProfileMakeR, machine learning, non-constitutive volatile, Random Forest

## Abstract

*Narcissus* flowers are used as cut flowers and to obtain high quality essential oils for the perfume industry. As a winter crop in the Mediterranean area, it flowers at temperatures ranging between 10 and 15°C during the day and 3–10°C during the night. Here we tested the impact of different light and temperature conditions on scent quality during post-harvest. These two types of thermoperiod and photoperiod. We also used constant darkness and constant temperatures. We found that under conditions of 12:12 Light Dark and 15-5°C, *Narcissus* emitted monoterpenes and phenylpropanoids. Increasing the temperature to 20°-10°C in a 12:12 LD cycle caused the loss of cinnamyl acetate and emission of indole. Under constant dark, there was a loss of scent complexity. Constant temperatures of 20°C caused a decrease of scent complexity that was more dramatic at 5°C, when the total number of compounds emitted decreased from thirteen to six. Distance analysis confirmed that 20°C constant temperature causes the most divergent scent profile. We found a set of four volatiles, benzyl acetate, eucalyptol, linalool, and ocimene that display a robust production under differing environmental conditions, while others were consistently dependent on light or thermoperiod. Scent emission changed significantly during the day and between different light and temperature treatments. Under a light:dark cycle and 15-5°C the maximum was detected during the light phase but this peak shifted toward night under 20-10°C. Moreover, under constant darkness the peak occurred at midnight and under constant temperature, at the end of night. Using Machine Learning we found that indole was the volatile with a highest ranking of discrimination followed by D-limonene. Our results indicate that light and temperature regimes play a critical role in scent quality. The richest scent profile is obtained by keeping flowers at 15°-5°C thermoperiod and a 12:12 Light Dark photoperiod.

## Introduction

Plants are able to produce and emit a high variety of volatile organic compounds (VOCs). Plant volatiles play several and complex roles in stress response and chemical communication, including pest repellence, herbivore deterrence, pollinator attraction, and plant-plant interaction (Jürgens et al., 2000; Matsui, 2006; Niinemets et al., 2013; van Dam and Bouwmeester, 2016). Principal volatile compound classes include fatty acid derivatives, terpenoids, and benzenoids/phenylpropanoids, synthesized in four major pathways: methylerythritol phosphate, mevalonic acid, lipoxygenase, and shikimate/phenylalanine (Dudareva et al., 2013; Muhlemann et al., 2014; Nagegowda, 2018).

Interestingly, plants emits a mix of compounds and their respective amount determines a particular aroma blend (Füssel et al., 2007; Weiss et al., 2016). Moreover, scent profiles differ between plant organs and tissues, such as leaves and petals, but also among species, genotypes and even life span (Jürgens et al., 2002; Dötterl et al., 2005; Majetic et al., 2014). In addition, the synthesis and emission of volatile compounds rely on biotic and abiotic factors (Peñuelas and Llusià, 2001; Loreto and Schnitzler, 2010). Temperature affects floral scent emission in a variety of species such as Petunia, *Osmanthus* or *Lilium* (Sagae et al., 2008; Hu et al., 2013; Fu et al., 2017). This indicates that both growing conditions and managing temperatures during post-harvest may affect the actual scent profile. Light intensity and light quality or spectral distribution also play a role in coordinating scent emission (Hu et al., 2013; Chuang et al., 2017). The number of released terpenoids, alcohols or aromatic compounds increases with light intensity in corn plants and *Lilium* “Siberia” (Gouinguené and Turlings, 2002; Hu et al., 2013). Compared with white light, red, and far red lighting cause increased release of phenylpropanoids/benzenoids compounds in petunia flowers (Colquhoun et al., 2013).

Sensorial quality of fruits and cut flowers such as aroma and flavor, is important to consumers. Both aroma and flavor depend on genotype, crop management, culture practices, maturity but also post-harvest management. Several methods can modify and improve the scent quality and the content in sugars and phenolic compounds. Post-harvest handling include controlled atmospheres (Lopez et al., 2000), UV-B irradiation (Hagen et al., 2007) and storage temperature, which has been widely studied in fruits and vegetables. Tomato and pineapple fruit for example, show a higher accumulation of aromatic compounds as esters and ketones under elevated temperatures (Maul et al., 2000; Liu and Liu, 2014). Moreover, a high temperature is not optimal for long distance shipping or long storage, which typically require low temperatures and is carried out in darkness. Low temperature prevents fruit ripening and pathogen proliferation but it may result in chilling injuries and loss of flavor and aroma quality (Imahori et al., 2008; Weiss and Egea-Cortines, 2009; Tietel et al., 2012).

Cut flowers are also shipped and stored in modified atmospheres and/or low temperatures. As described in fruits and vegetables, low temperature tolerance vary among species (Redman et al., 2002). Low temperature has undesired effects as chilling injury symptoms including loss of coloration and flower malformation (Joyce et al., 2000; Bunya-atichart et al., 2004). In addition to this, inadequate post-harvest handling may alter enzymatic activity and secondary metabolism, affecting aroma volatile composition.

Floral volatiles and their distilled oils from *Narcissus* sp. are important in the perfume and chemical industry (Remy, 2004). The flowering of narcissi like other geophytes is activated by cold and flowers in winter in Mediterranean environments (Rees and Wallis, 1970; Rees and Hanks, 1984; González et al., 1998). A correct post-harvest technical knowledge is key to preserve the quality of cut flowers. Pre-harvest management also has an impact in essential oil yield and composition (Perry et al., 1999; Angioni et al., 2006). In the last years, several researchers have analyzed the relationship between environment and volatiles quality and quantity. Terpenes, an abundant group of VOCs, show a positive correlation between emitted amount and temperature and light (Staudt and Lhoutellier, 2011; Chen et al., 2020). Another important aspect is scent composition, which changes in response to high temperature and drought stress (Haberstroh et al., 2018). In relation to environmental factors, several studies address climate change and its possible impact on plant volatiles (Yuan et al., 2009). The effect of climate warming seemed to differ in terms of emitted amount, but has a noticeable effect on scent composition (Faubert et al., 2012; Schollert et al., 2015). Changes in aroma blend may alter several biological functions as pollinator attraction, plant defense or plant-plant communication (Farré-Armengol et al., 2013). Therefore, environmental conditions, both in pre-harvest and post-harvest conditions, play a critical role on the synthesis and emission of volatiles.

While metabolomic studies of fruits often include complete metabolite profiles, the number of studies describing the environmental effects on complete metabolomes of flowers has been performed in very few species (Verdonk et al., 2003; Cheng et al., 2016). The aim of the present work was to define the effect of environmental conditions during post-harvest on the quantity and composition of *Narcissus* sp. (Amaryllidaceae) scent. Our results indicate a significant effect of light regimes, and specially temperature, on scent quantity and profiles.

## Materials and Methods

### Plant Material and Experimental Design

**FIGURE 1:**
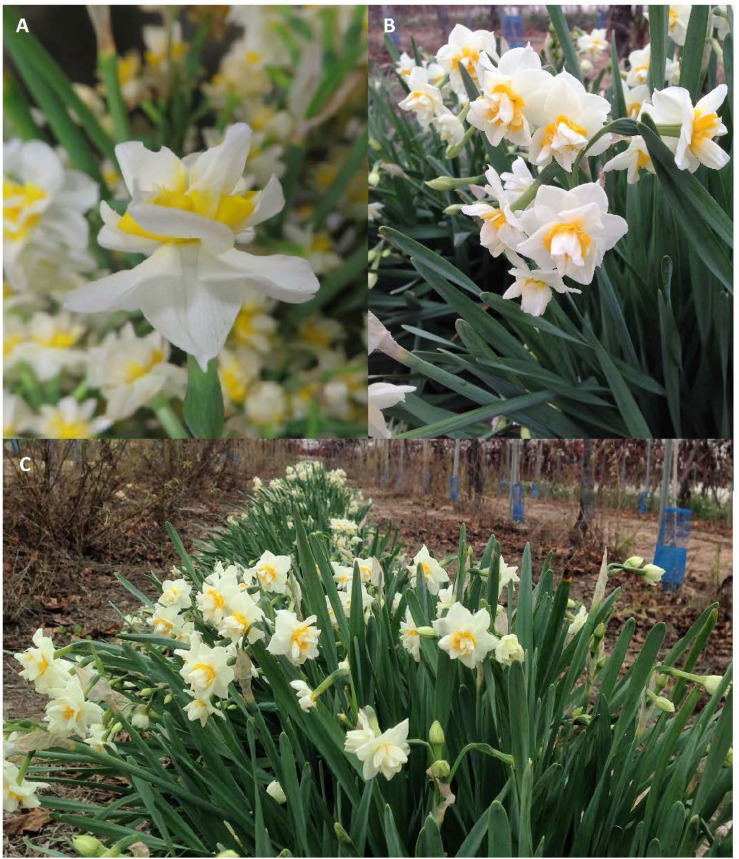
Flowers of *Narcissus* sp. “Double flower” used in the study. **(A)** Close up of double flower. **(B)** Plants at harvesting point. **(C)** Overview of Narcissus growing as intercrop between organic grape vines.

*Narcissus* (Amaryllidaceae) is a group of perennial bulbiferous widely distributed in the Mediterranean basin. The number of species is still unclear. The presence of several hybrids makes it difficult to identify narcissi species as well as define their distribution (Santos-Gally et al., 2012; Marques et al., 2017). In Spain, at least 25 species have been described as well as different hybrids (Aedo and Herrero, 2005). We used a local *Narcissus* cultivar variety called “double flower” owned by Cooperativa las Cabezuelas (Figure 1A). The variety was originally isolated by Matías Águila Noguera in 1960, founder of Las Cabezuelas, and has been reproduced by bulbs ever since (DÁ, unpublished observation). Plants grew as intercrop between organic grapevines in Las Cabezuelas (Figures 1B,C), located in Alhama de Murcia (Spain) as described before (Ruíz-Ramón et al., 2014). Narcissi were harvested at flower opening, coinciding with anthesis, and cut flowers were brought to the lab maintained with the stalks in water until volatile sampling. As narcissi flowers have a short lifespan, we performed the volatile sampling in the first 24–36 h after harvesting. Harvesting was the moment of cutting in the field roughly at 0830. Floral stalks were kept on water glasses and brought to the lab at 1800. Sampling was performed during the winter of 2015 and 2016. We selected flowers without any visual damage. Each flower was weighted before sampling and placed into a growth chamber. Flowers were kept in the chamber in the initial conditions 1 h before the first twister was introduced. We collected volatiles under six different conditions: four assays were conducted under light:dark conditions and two under constant darkness (see below). Constant darkness simulated storage and/or shipping conditions. In Alhama de Murcia in winter (December-February), average temperature is 11.1 ± 5.2°C, with minimum of 4 and maximum of 15.7°C. We decided to study the aroma emission under a range of temperature similar to field conditions (15-5°C) and simulate a hypothetical warm winter day (20-10°C). Furthermore, we also analyzed the effect of constant temperature, typical of post-harvest conditions on scent emission and aroma profile. Then, experimental conditions were defined as follow, comparing three pairs of treatments: First, we analyzed the scent emission under a 12 h light and 12 h dark cycle (12LD) and two different temperature regimens: 20-10°C and 15-5°C. The highest temperature coincided with the light period whereas the lowest temperature, corresponded to the dark phase. We defined the 12LD and 15-5°C group as control conditions. The second experiment consisted in continuous darkness (12DD) and two thermoperiods, 20-10°C and 15-5°C. Finally, in the last experiment flowers were sampled under a 12LD cycle with constant temperature of 20°C and 5°C, respectively. For light:dark experiments, we defined *zeitgeber* time 0 (ZT0) as the time when lights turned on. For constant darkness experiments, we defined ZT0 as the time when high temperature cycle started.

### Analysis of Volatile Compounds

We used five narcissi cut flowers for each experiment. We introduced one cut flower during 24 h into a beaker containing a 4% sucrose solution, which was located into a 2l desiccator. Each desiccator consisted in a glass container, which we cleaned before and after sampling with 100% ethanol. Volatiles were collected by SBSE magnetic stir bar sorptive extraction, GERSTEL, coated with polydimethylsiloxane (PDMS) Twister, as they absorb volatile non-polar compounds optimally (Bicchi et al., 2005). Twisters were previously conditioned, by incubating at 40°C and ramping to 300°C at 25°C/min and maintaining at 300°C for 20 min. In each container, we introduced a paper clip in order to attach a stir bar. We used empty desiccators (without cut flowers) as negative controls.

We analyzed volatile emission at different time points during the light and dark period or subjective day and night. Two time points corresponded to the subjective day and two to the subjective night, ZT0, ZT4, ZT16, and ZT22 for constant darkness and constant temperature groups, and ZT4, ZT16, ZT22, and ZT24 for light;dark and cycling temperature. Twisters were left inside the desiccators for a period of 2 h.

Volatiles were separated and identified as described by Manchado-Rojo et al. (2012). Briefly, we used a 6890 gas chromatograph coupled to a 5975 inert XL mass selective detector (Agilent Technologies) with a thermal desorption unit, a cooled injector system and a multi-purpose sampler (MPS2, GERSTEL). We used a HP-5MS capillary column (30 m length, 0.25 mm internal diameter and 0.25 m film) in constant pressure, using helium as carrier gas. The temperature was increased from 50 to 70°C (5°C per min) held 1 min and then increased to 240°C (10°C per min) and held 15 min. Twister bars were desorbed using an initial temperature of 40°C ramping to 150°C (100 C per min) and a holding time of 5 min. The transfer temperature was 300°C. The desorbed compounds were cryo-focused in the cooled injector system inlet at -100 C. Finally, volatiles were transferred into the column by heating the CIS4 inlet at a rate of 10°C sec^–1^ to 150°C with a holding time of 3 min.

In those cases when we did not have commercial standards to identify properly VOCs, we tentatively identified compounds using Willey10th-NIST11b (Agilent Technologies, Wilmington, United States). The integrated peak area of every identified volatile was normalized by dividing it by the flower fresh weight (Ruiz-Hernández et al., 2018).

### Data Analysis

We considered that compounds that appeared in more than 70% of the replicates, with an average quality match with mass-spectra library over 80%, could be considered as part of the constitutive scent emission of narcissi flowers under each treatment. We obtained the scent profiles by using the R library “gcProfileMakeR” version 2.2.2 (Pérez-Sanz et al., 2020). We set the parameters pFreqCutoff (minimum frequency cutoff, percentage of samples that emit a volatile) to 0.70 and qcutoff (compound quality, expressed as percentage) to 80. We also applied an initial filter to remove compounds by their CAS number, as siloxanes that may derive from stir bars (Montero et al., 2005; Supplementary Table 1). In order to visualize the data, we classified the selected compounds based on their contribution to the scent profile, in two groups. Major volatiles contributed to the scent profile above 2% and remained compounds comprised the minor volatile group (see section “Results”).

The study of biogenic volatiles can generate large amounts of data. Tools such as the R library gcProfileMakeR determines the core profile and non-constitutive profile of a set of samples (Pérez-Sanz et al., 2020). These profiles can be analyzed with Machine Learning methods. They are useful to identify volatile signatures, which allow recognizing different organisms (Ranganathan and Borges, 2010). Algorithms such as Random Forest (Breiman, 2001) can identify the compounds that can be used for classifying between different sets of samples.

We used the Random Forest algorithm to identify those volatiles that can be used for classifying the different treatments of photoperiod and thermoperiod (Breiman, 2001). This analysis was performed by the R package “randomForest” (Liaw and Wiener, 2002). We merged all sampling time points per replicate, photoperiod and thermoperiod conditions.

To analyze the effects of experimental conditions and sampling time points on scent quantities, we performed a Kruskall–Wallis test followed by a Dunn test as *post hoc* analysis, implemented in stats and dunn.test packages (R version 3.6.1) (Dinno, 2017).

We used a principal component analysis (PCA) to explore differences across experimental treatments. The complete set of volatiles for each replica point was used as input. The analysis was performed in R using the prcomp function of the stats package, and plotted with ggfortify (Tang et al., 2016).

## Results

### Scent Profiles Are Affected by Photoperiod and Thermoperiod

**FIGURE 2:**
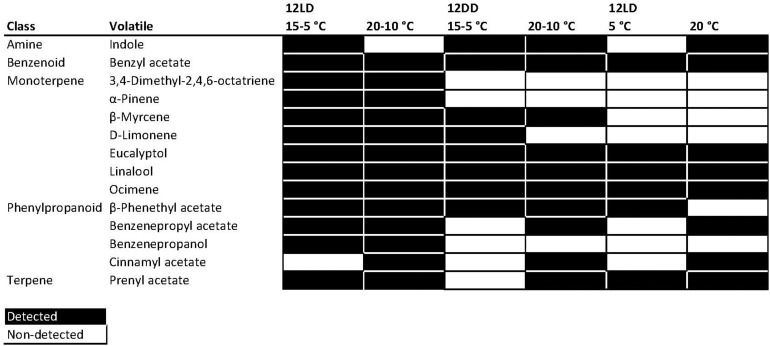
Heat map of volatiles emitted by Narcissus across experimental conditions. First column shows the chemical abstract service (CAS) number of the volatiles. 12LD indicates a 12-h light:dark cycle and 12DD constant darkness. Black indicates a detected compound and white an undetected compound.

Narcissi cut flowers showed a complex profile. The number of identified compounds by GC-MS varied among conditions and daytime. We identified in total 73 compounds with a quality higher than 80% (Supplementary Figure 1 and Table 1). For every experimental group, we filtered the list of volatiles using the R library “gcProfileMakeR” (Pérez-Sanz et al., 2020). We selected the volatiles emitted by more than 70% of the samples with a minimum quality of 80% for every experimental condition labeling them as constitutive volatiles. We obtained a list of 68 volatiles present in the six different experimental conditions. They were divided in constitutive where we found 14 VOCs and non-constitutive comprising 64 VOCs (Figure 2 and Supplementary Figure 1). The minimum quality was 87% for myrcene. These compounds comprised mainly monoterpenes and phenylpropanoids (Table 1). The rest of volatiles found were non-constitutive and were found in less than 70% of the samples for a given environmental condition (Supplementary Figure 1). We defined the group of cut flowers sampled under 12h light: 12h dark (12LD) and 15-5°C as the control group. These are the typical day/night temperatures found in the field during December/January when they flower (Viti et al., 2010).

**TABLE 1:** Selected volatile organic compounds from Narcissus cut flowers.

CAS	RT	Quality	Volatile	Class
000120-72-9	11.982	96.9	Indole	Amine
000140-11-4	9.42	97.3	Benzyl acetate	Benzenoid
007785-26-4	5.011	95.8	α-Pinene	Monoterpene
000123-35-3	6.311	95.1	β-Myrcene	
005989-27-5	7.134	97.3	D-Limonene	
000470-82-6	6.858	98.3	Eucalyptol	
003779-61-1	7.577	98.0	Ocimene	
000078-70-6	8.592	96.0	Linalool	
057396-75-5	9.158	96.2	3,4-Dimethyl-2,4,6-octatriene	
000103-45-7	11.22	89.5	β-Phenethyl acetate	Phenylpropanoid
000122-97-4	10.973	95.9	Benzenepropanol	
000122-72-5	13.134	90.1	Benzenepropyl acetate	
000103-54-8	14.196	96.7	Cinnamyl acetate	
001191-16-8	4.744	93.7	Prenyl acetate	Terpene

*We detected these compounds in at least the 70% of the samples, in one or more experimental groups, with a minimum quality of 80%. CAS chemical abstracts service registry number, RT retention time expressed in minutes, Quality average match quality expressed as a percentage.*

We found 13 different compounds under 12LD and 15-5°C thermoperiod (Figure 2). When we mimicked the situation of a warm winter, with 12LD and 20-10°C, we found indole, not present in the control conditions, while cinnamyl acetate became a non-constitutive component of the profile (Figure 2 and Supplementary Figure 1). Plants under constant darkness (12DD) with thermocycle emitted 10 VOCs at 20-10°C and 8 volatiles at 15-5°C. In contrast, flowers under a 12LD and constant temperature showed a lower complexity. We only detected 8 and 6 volatiles at 20 and 5°C, respectively. We found the highest complexity, defined as number of detected VOCs, under cycling light and thermoperiodic conditions.

As the number of detected compounds varied among experimental conditions, we defined four groups. The first group comprised four robustly emitted compounds that were detected regardless of light or temperature conditions. These were the monoterpenes eucalyptol, linalool and ocimene, and the benzenoid benzyl acetate. Thus, these compounds can be considered the common chemical scent profile of this *Narcissus* variety. A second group contained 3,4-dimethyl-2,4,6 octatriene, pinene, and benzenepropanol, that were only detected under cycling conditions of light and temperature. The monoterpenes myrcene and limonene were not detected under a constant temperature of 5°C or 20°C (Figure 2). The remaining compounds, indole, phenethyl acetate, and prenyl acetate, were variable. Although many compounds appeared under several conditions, their amount or contribution varied (Figure 2, see below). We can conclude that the scent profile in Narcissus comprises a set of volatiles robustly produced under different environmental conditions, a second set that requires light cycling conditions, a third set that depends on thermoperiod, and a last one with variable behavior.

**FIGURE 3:**
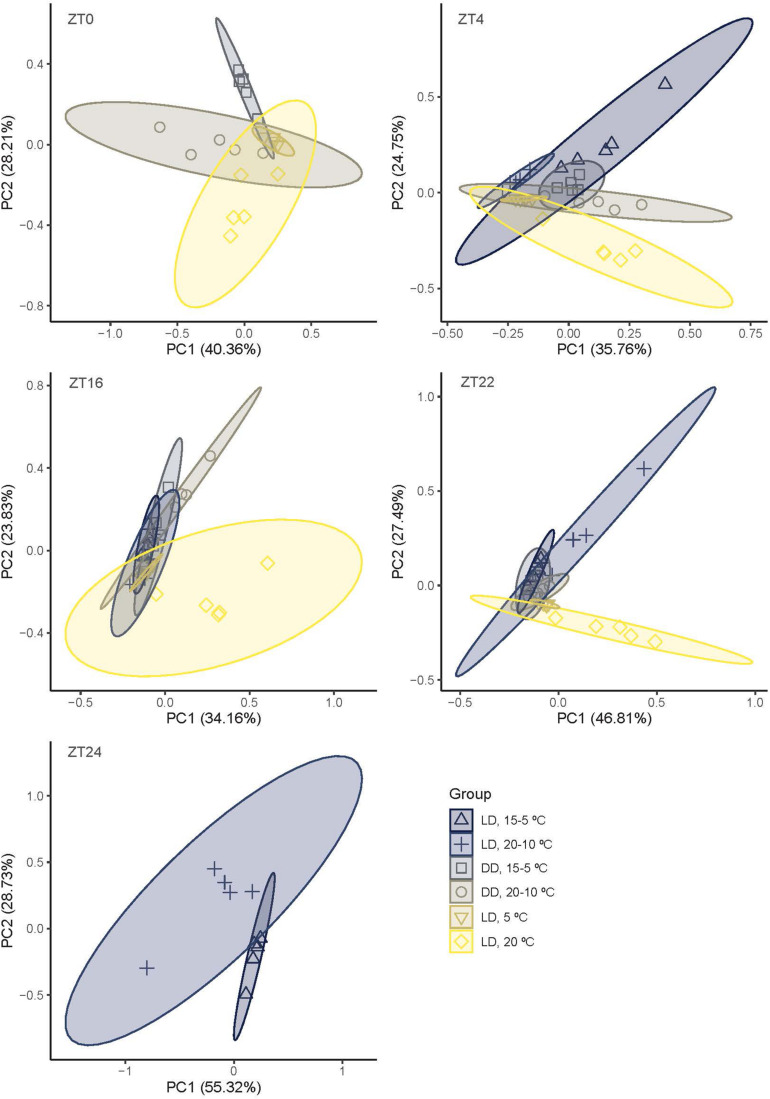
Principal component analysis (PCA) based on narcissi emitted volatiles at five time points (ZT, *zeitgeber* time) under six different light:dark and temperature conditions. LD, light:dark; DD, constant darkness; PC, principal component.

We explored the differences in qualitative changes through the sampling periods to identify possible clusters. We used PCA at different time points and comparing three pairs of treatments (Figure 3). The two first principal components, PC1 and PC2, explained the 68.57% of the variation (ZT0), 60.51% (ZT4), 57.98% (ZT16), 74.29% (ZT22), and 84.05% (ZT24) (Figure 3 and Supplementary Table 2). Groups sampled under 12LD/15-5°C, 12DD/15-5°C, and 12LD/5°C showed a similar pattern and clustered together. In contrast, the aroma blend of narcissi sampled under 12LD and constant 20°C differed from other narcissi groups. We also observed variations across time and conditions. At ZT0, 12DD/15-5°C and 12LD/5°C patterns were similar and clustered together whereas 12DD/20-10°C and 12LD/20°C differed. At ZT4, narcissi under 12LD/15-5°C and 12LD/20-10°C, constant darkness and constant temperature showed a similar distribution. The 5°C constant temperature treatment caused an extremely compact cluster. It was centered with most profiles, as it comprises the four constitutive volatiles found under all conditions. As mentioned previously, at ZT16 and ZT22, all experimental conditions showed a similar pattern except those narcissi that were sampled under 12LD and 20°C. Finally, at ZT0 12LD/15-5°C and 12LD/20-10°C aroma patterns were different (Figure 3).

We found that the importance of each volatile (expressed as loading factors) to the narcissi aroma changed along the day (Supplementary Table 2).

The first principal component revealed that the most important volatiles were ocimene at ZT0, ZT4, benzyl acetate at ZT16 and ZT22, and pinene at ZT24. The volatiles limonene at ZT0 and ZT4, ocimene at ZT16 and ZT24 and phenethyl acetate at ZT22 contributed positively to the PC2 (Supplementary Table 2). Two of these volatiles, benzyl acetate and ocimene, were constitutively emitted. In contrast, pinene was absent in narcissi sampled under constant darkness and constant temperature, phenethyl acetate was not detected at constant 5°C and pinene emission was variable and detected under 12LD/15-5°C, 12LD/20-10°C, and 12DD/20-10°C (Figure 2). All these results suggested that temperature, light and daytime determined the aroma blend of narcissi cut flowers as well as its emission pattern.

### Effect of Thermoperiod and Photoperiod on Quantitative Changes in Scent Profiles

**FIGURE 4:**
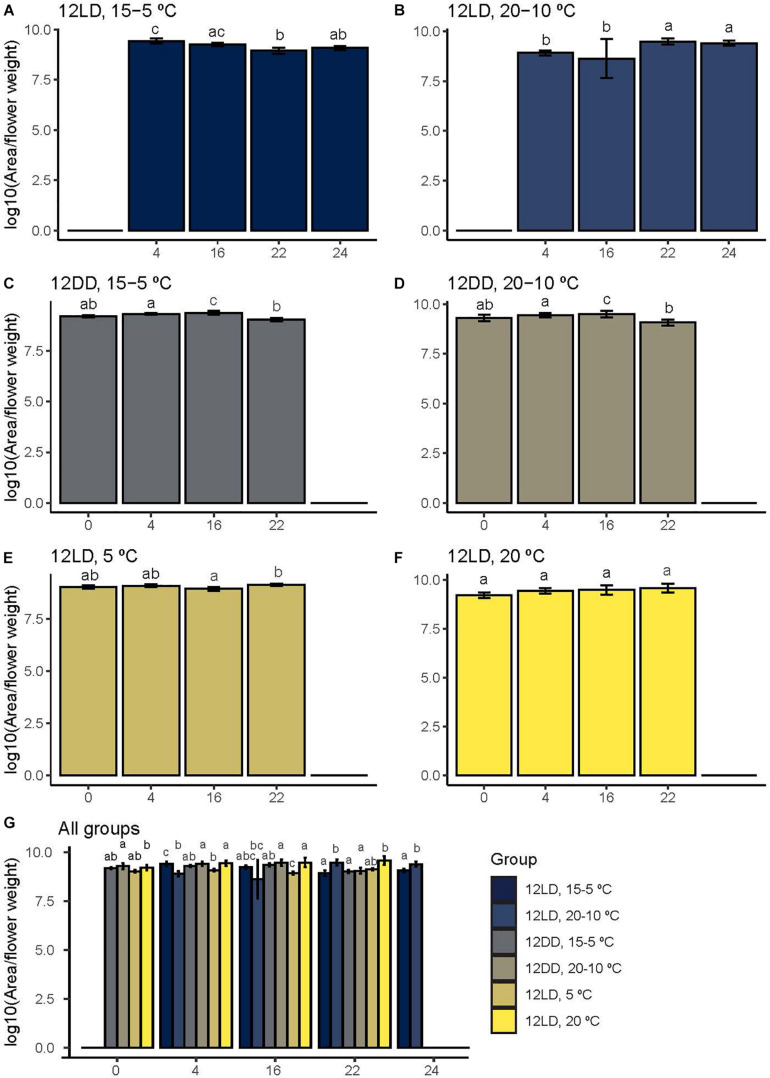
Narcissi flower total scent emission under six different light:dark and temperature conditions. Volatiles were sampled at four time points (ZT). To detect scent emission patterns, we analyzed each group along time **(A–F)**. We also compared the emitted amount under six experimental conditions at each time point **(G)**. Each bar represents the average ± standard deviation of five flowers. Letters over bars indicate differences among time points **(A–F)** and groups **(G)** (Dunn’s test, see Tables 2, 3), LD, light:dark; DD, continuous dark; fw, fresh weight.

The contribution of every volatile to the floral aroma determines a specific aroma blend. A previous study characterized the floral aroma of several *Narcissus* species (Dobson et al., 1997). The scent profile obtained in the present work was dominated by monoterpenes and phenylpropanoids. However, amines and a benzenoid were also detected (Figure 2 and Supplementary Figure 1; Ruíz-Ramón et al., 2014). We divided narcissi in three experimental groups and each group consisted in two different light:dark and temperature conditions, sampling volatiles at four time points. As we expected to observe changes along time but also among experimental treatments, we performed two different analysis. First, we analyzed each group and each pair in order to determine if the release of volatiles increased or decreased at different times of the day/night period (Figures 4A–F and Table 2). This data was log-transformed.

**TABLE 2:** Analysis of narcissi volatile emission at four time points under different light and temperature conditions.

Group	Comparison (ZT)								
	0–4	0–16	0–22	4–16	4–22	4–24	16–22	16–24	22–24
12LD, 15-5°C	–	–	–	0.372	0.003	0.026	0.024	0.163	0.364
12LD, 20-10°C	–	–	–	0.021	0.708	0.043	0.010	0.668	0.016
12DD, 15-5°C	0.021	0.043	0.668	0.708	0.010	–	0.016	–	–
12DD, 20-10°C	0.342	0.223	0.242	0.708	0.026	–	0.017	–	–
12LD, 5°C	0.403	0.299	0.174	0.074	0.454	–	0.017	–	–
12LD, 20°C	N.S.	N.S.	N.S.	N.S.	N.S.	–	N.S.	–	–

*For each group, all pairwise multiple comparison were calculated using a Dunn’s test. 12LD, 12h light:12h dark; 12DD, constant darkness; ZT, *zeitgeber* time; N.S., no significant differences.*

The time series analysis revealed different scent emission patterns (Figures 4A–F and Table 2). Under control conditions, 12LD and 15-5°C, the maximum scent release occurred during the light period at ZT4 and decreased significantly at night (Dunn’s test *p* < 0.05, Figure 4A). In contrast, under 12LD and 20-10°C, the scent emission increased progressively and significantly along the light-dark period, showing opposite (Figure 4B). Under constant darkness, and irrespective of temperature regimes, the emission displayed a delayed pattern compared to control (Figures 4C,D). Indeed, the control maximal emission occurred at ZT4 while constant dark peaked at ZT16 and declined significantly thereafter (Dunn’s test *p* < 0.05, Table 2 and Figures 4C,D). Narcissi floral emission sampled under constant 5°C decreased at midnight (ZT16), whereas under constant 20°C revealed a somewhat stable emission (Figures 4E,F).

In order to assess the effects of light and temperature on overall scent emission we compared the emitted amount at each time point (Figure 4G and Table 3). We analyzed each pair of experimental conditions, e.g., constant 5 and 20°C, and we compared all groups among them. At ZT0 we did not observe differences among narcissi pairs (*p* > 0.05). Moreover, the emitted amount under constant darkness and constant temperature differed and we observed the lowest emission under constant 5°C (Figure 4G and Table 3). At ZT4, the emitted amount differed between two experimental pairs: 12LD/15-5°C and 12LD/20-10°C and constant 5 and 20°C (*p* < 0.05). Comparing all groups we observed that flowers under 12LD/20-10°C and 12LD/5°C displayed the lowest emission (Figure 4G). We observed a similar pattern at ZT16. The two groups of narcissi sampled under constant temperature showed a significant difference in emitted amounts (*p* < 0.05) whereas remaining pairs did not change. When we compared all groups, we found that the lowest emission corresponded to 12LD/20-10°C and 12LD/5°C, as observed at ZT4 (Figure 4G and Table 3). At ZT22, we only observed difference in the pair 12LD/15-5°C and 12LD/20-10°C (*p* < 0.05, Figure 4). Furthermore, the emission under 12LD/20-10°C and 12LD/20°C was higher compared with other groups (Figure 4G and Table 3). Finally, at ZT24 the pair sampled under LD and cycling temperature displayed a significant difference between 15-5°C and 20-10°C, with a highest emission under 20-10°C (Figure 4G and Table 3).

**TABLE 3:** Analysis of narcissi volatile emission under different light and temperature conditions.

Comparison		ZT0	ZT4	ZT16	ZT22	ZT24
12LD, 15-5°C	12LD, 20-10°C	–	0.005	0.214	0.214	0.020
	12DD, 15-5°C	–	0.406	0.598	0.598	–
	12DD, 20-10°C	–	0.971	0.258	0.258	–
	12LD, 5°C	–	0.027	0.103	0.103	–
	12LD, 20°C	–	1	0.251	0.251	–
12LD, 20-10°C	12DD, 15-5°C	–	0.051	0.065	0.065	–
	12DD, 20-10°C	–	0.006	0.012	0.012	–
	12LD, 5°C	–	0.563	0.742	0.742	–
	12LD, 20°C	–	0.011	0.013	0.013	–
12DD, 15-5°C	12DD, 20-10°C	0.453	0.422	0.591	0.591	–
	12LD, 5°C	0.196	0.246	0.026	0.026	–
	12LD, 20°C	0.641	0.442	0.586	0.586	–
12DD, 20-10°C	12LD, 5°C	0.039	0.029	0.006	0.006	–
	12LD, 20°C	0.659	1	0.943	0.943	–
12LD, 5°C	12LD, 20°C	0.117	0.033	0.010	0.010	–

*For each time point, all pairwise multiple comparison were calculated using a Dunn’s test. 12LD, 12h light-12h dark; 12DD, constant darkness; ZT, *zeitgeber* time.*

Although emission patterns changed among groups (Figures 4A–F, see above), narcissi sampled under constant temperatures showed important differences, except at ZT0. Narcissi scent emission under constant 20°C was higher than at 5°C (Figure 4G and Table 3).

We can conclude that floral scent emission is strongly affected by temperature regimes in *Narcissus*, while light plays a lesser role.

### Effect of Thermoperiod and Photoperiod on Qualitative Changes in Scent Profiles

**FIGURE 5:**
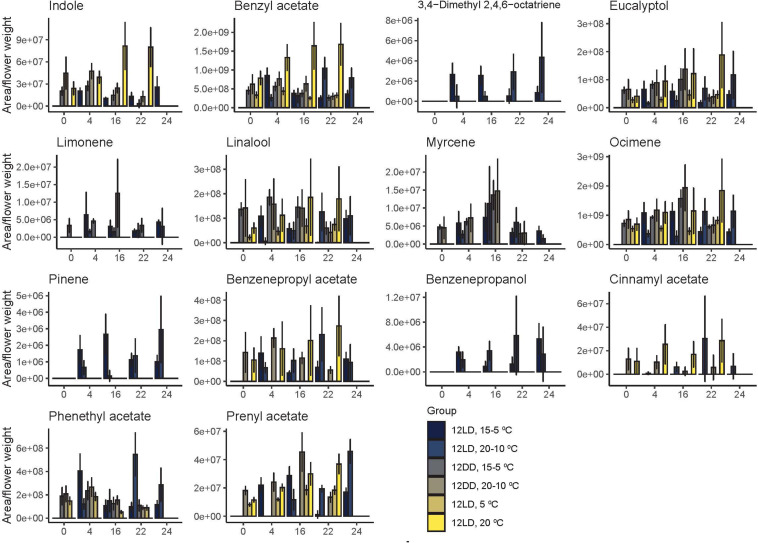
Daily emission of narcissi volatiles. Each bar represents the average emission ± standard deviation of five flowers at four different time points (*zeitgeber* time, ZT).

The observed changes in scent emission among narcissi groups (Figure 4G and Table 3) could be the result of different emission patterns (Figures 4A–F and Table 2), the effect of temperature on enzymatic activity, storage, and/or release of VOC but also may be related to the absence of certain volatiles (Figure 1). We examined the emission of every volatile and its contribution to the narcissi scent (Figure 5).

Under cycling light and both temperature cycles, 15-5°C and 20-10°C, we found a first group of volatiles with maximum emissions during the light period. These included 3,4-dimethyl 2,4,6-octatriene, eucalyptol, and limonene. In contrast, a second group reached their maximum quantity at night. It comprised benzenpropyl acetate, benzenepropanol, myrcene, and prenyl acetate. Other compounds such as ocimene, did not follow a clear pattern. Under a 15-5°C cycle, the highest emission of ocimene occurred during the light period whereas under 20-10°C, this volatile displayed its maximum amount at night (Figure 5).

Flowers sampled in constant darkness with cycling temperature, 15-5°C and 20-10°C showed two patterns. The highest amount of indole, benzyl acetate, benzenepropyl acetate, linalool, and phenethyl acetate were found at ZT4. In contrast, we found the highest amount of eucalyptol, limonene, myrcene, ocimene, cinnamyl acetate, and prenyl acetate during the subjective night, at ZT16 and ZT22 (Figure 5).

As mentioned above, we detected the highest quantity of volatiles under a light:dark cycle and a constant temperature of 20°C (Figure 4). Interestingly, the maximum amounts of all volatiles appeared during the dark period, at ZT16 or ZT22. This pattern was similar under a light:dark cycle with constant temperature of 5 C. Indeed, the emitted amount of eucalyptol, linalool, ocimene, and prenyl acetate increased during the dark period (Figure 5). In contrast, benzyl acetate and phenethyl acetate showed their maximum emission during the light period. Altogether, these results revealed that the maximum levels of a volatile depended on time of the day, light and temperature conditions. Furthermore, narcissi sampled at constant 5° and 20°C revealed a lower number of volatiles, compared to control group (Figure 2). In this case seemed that temperature but not the number of emitted compounds determine the scent intensity (Figures 4, 5).

### Random Forest Classification

The scent profiles obtained appeared to be highly divergent between the different temperature and light regimes. We investigated if the diverging profiles could be classified as different using Machine Learning algorithms. In order to identify the volatiles, among the 14 selected compounds, which can be useful for classification, we used the Random Forest algorithm. The model classified correctly all the samples except one of LD continuous temperature of 20°C that was classified as continuous 5°C (Table 4). The obtained out-of-bag error (OOB) was 3.33%. This analysis ranked the volatiles based on mean decreasing accuracy (MDA) (Table 5). The five most important volatiles to classify between light:dark and temperature conditions were indole, limonene, cinnamyl acetate, myrcene, and benzenepropyl acetate (Table 5). These results confirmed that the scent profile was different among analyzed conditions. However, and as mentioned above, constant temperatures, typical of post-harvest conditions, caused the most drastic changes in scent profiles.

**TABLE 4:** Random forest confusion matrix showing the observed and the predicted groups among light:dark and temperature cycles.

Observed	Predicted						Error
	DD, 15-5°C	DD, 20-10°C	LD, 15-5°C	LD, 20-10°C	LD, 20°C	LD, 5°C	
DD, 15-5°C	5	0	0	0	0	0	0
DD, 20-10°C	0	5	0	0	0	0	0
LD, 15-5°C	0	0	5	0	0	0	0
LD, 20-10°C	0	0	0	5	0	0	0
LD, 20°C	0	0	0	0	4	1	0.2
LD, 5°C	0	0	0	0	0	5	0

*Every group consisted in five replicates. LD, light:dark; DD, constant darkness.*

**TABLE 5:** Importance ranking of volatile organic compounds (VOCs) for classifying narcissi by random forest algorithm, based on mean decreasing accuracy (MDA).

Rank	VOCs	Class	MDA
1	Indole	Amine	14.56
2	D-Limonene	Monoterpene	13.52
3	Cinnamyl acetate	Phenylpropanoid	12.81
4	β-myrcene	Monoterpene	12.65
5	Benzenepropyl acetate	Phenylpropanoid	12.57
6	Benzyl acetate	Benzenoid	11.33
7	Prenyl acetate	Terpene	11
8	β-Phenethyl acetate	Phenylpropanoid	10.92
9	Benzenepropanol	Phenylpropanoid	10.35
10	3,4-Dimethyl 2,4,6-octatriene	Monoterpene	10.25
11	α-Pinene	Monoterpene	10.04
12	Ocimene	Monoterpene	3.72
13	Linalool	Monoterpene	3.53
14	Eucalyptol	Monoterpene	0.83

## Discussion

The absolute emitted amounts and specific composition of fragrances are two factors, both for the industry and at the ecological level. Indeed, industrial quality of essential oils is based on the specific composition in both qualitative and quantitative terms (Nejad Ebrahimi et al., 2008; Nabiha et al., 2009). Scent composition is an important component of breeding in ornamentals such as *Chrysanthemum* or *Iris* (Sun et al., 2015; Yuan et al., 2019). As ambient conditions may affect scent emission at the metabolic level and/or aroma accumulation and vaporization (Hu et al., 2013; Farré-Armengol et al., 2014; Cna’ani et al., 2015), we have studied the effect of light and temperature on *Narcissus* scent.

Scent emission is also related to flower development, and stops rapidly upon floral senescence (Dudareva et al., 2000; Weiss et al., 2016). As the *Narcissus* variety used in this study showed a very short life, we had to do single day tests. The mistletoe flower scent complexity decreases along its lifetime, volatiles such as nonanal and farnesene were not present in all flower stages. At the same time, the emitted amount of ocimene increases during the lifespan of flowers (Quintana-Rodríguez et al., 2018). These modifications of aroma occur within days, suggesting changes in volatile biosynthesis, accumulation and release, and may be considered in post-harvest handling. Experiments in snapdragon and wild relatives have shown a stable scent profile within a span of 3 to 5 days, indicating that there is a genetic component defining floral lifespan (Weiss et al., 2016). This may also play an important role in scent emission and stability.

Several flowering species including rose, snapdragon or tobacco display a robust circadian pattern (Kolosova et al., 2001; Raguso et al., 2003; Picone et al., 2004). The robust circadian scent oscillation is maintained by *PhLHY, NaZTL, PhCHL, AmLHY*, and *PhGI1* (Fenske et al., 2015; Yon et al., 2015; Terry et al., 2019a, b; Brandoli et al., 2020). As coordination of circadian scent emission can be considered a downstream process of the clock, it is important to determine if daily changes in scent emission respond to drastic modifications in environmental conditions such as those used in post-harvest, or as a result in natural changes in growing temperatures.

The daily changes in emission patterns and volatile quantities produces floral scent variation along the day (Barman and Mitra, 2019; Terry et al., 2019a, b). The aroma blend plays a role in pollinator attraction, plant defense and plant-plant interaction, among other (Heil and Ton, 2008; Schiestl, 2010; Ueda et al., 2012), and changes in the aroma can alter these functions. As previously reported, narcissi aroma consisted in monoterpenes, but also benzenoids and phenylpropanoids (Dobson et al., 1997; Ruíz-Ramón et al., 2014). Our results revealed that a modification in light:dark and temperature conditions can drastically alter the fragrance blend. We identified 14 volatiles organic compounds and four of them, benzyl acetate, eucalyptol, linalool, and ocimene, were constitutively emitted. Narcissi cut flowers also emitted phenylpropanoid and benzenoid volatiles. We can conclude that both benzenoid and monoterpene production is resilient to changes in environmental conditions, however, not all monoterpenes were robustly produced. Indeed, 3,4-dimethyl-2,4,6-octatriene, pinene, myrcene, and limonene emissions were controlled by light or temperature. As observed in monoterpene compounds, benzyl acetate was constitutive while all phenylpropanoids varied with environmental conditions. As the synthesis pathways of monoterpenes are shared (Dudareva et al., 2003; Byers et al., 2014), and benzenoids and phenylpropanoids are synthesized from phenylalanine (Boatright et al., 2004), an emerging hypothesis is that transport and/or synthesis maybe affected at specific biosynthetic steps. The effect of temperature in scent emission in *Narcissus* appears to be a common response in other geophytes.

Similar to stored terpenes in leaves, flowers can accumulate several volatile compounds, as petunia, that accumulates glycosylated phenylpropanoids, that are metabolized during the emission (Cna’ani et al., 2017). The metabolism of phenylpropanoids as well as its possible accumulation in narcissi flowers may explain the differences in emitted levels under different light and temperature conditions. Our results agree with those found in petunia, except under a constant temperature of 20°C. As terpenoids and phenylpropanoids show diverging patterns, we conclude that their synthesis or emission may have distinct genetic coordination.

The group comparison of temperature and light treatments suggested that the major differences found were imputable to constant temperatures. Indeed, emission maxima changed from day to night when comparing 15-5 to 20-10°C indicating a major impact of day night temperatures on overall scent emissions. The fact that continuous dark does not change the maximum emissions and constant temperatures caused a somewhat flat curve indicates that overall temperature has the highest impact in *Narcissus*. Changes in temperature have been shown to affect the quantity of emitted compounds in plants such as *Quercus ilex*, *Sonchus tenerrimus*, and *Lilium* sp. (Hu et al., 2013; Farré-Armengol et al., 2014). This pattern was especially remarkable under a constant temperature of 20°C, whereas the lowest amount of emitted VOCs was found under a constant temperature of 5°C (Figure 4). The timing of maximum emission under 12LD and 15-5°C occurred during the light phase but it was delayed to midnight under 20-10°C. Constant darkness shifted maximum emission to the end of the dark period and under constant temperature, narcissi scent emission tended to reach at the dark phase. These changes suggest that post-harvest conditions determine the emission pattern, indicating that light and temperature also control volatile accumulation and probably other secondary metabolites. As industrial post-harvest conditions tend to have fixed regimes of light and temperature, this aspect may be address in order to improve essential oil extraction.

As pre-harvest conditions are important in flower scent and essential oil quality and quantity (Angioni et al., 2006; Chen et al., 2020), growth under warm climate conditions may alter these properties. Some studies suggest that temperature is positively related with volatile emission (Faubert et al., 2010) including alteration of scent profiles (Faubert et al., 2012). However, some species appear to have stable emissions under changing environments such as the artic shrubs *Empetrum hermaphroditum* and *Cassiope tetragona* (Schollert et al., 2015). Temperature may regulate at different levels including metabolism, availability of substrates, enzyme activity and/or accumulation and release mechanisms (Oyama-Okubo et al., 2005; Farré-Armengol et al., 2013; Barman and Mitra, 2019). This may also depend on species, tissues and organs (Schollert et al., 2015; Jamieson et al., 2017).

Due to the complexity of plant volatilomes, classification methods such as principal component analysis, or machine learning algorithms as random forest are useful to identify patterns and to reduce the number of compounds that can be used to discriminate related species or varieties, among other procedures (Ranganathan and Borges, 2010; Dos-Santos et al., 2013; Brückner and Heethoff, 2017). In this study, narcissi under control conditions revealed more than 50 different identified compounds with a quality higher than 80% (Supplementary Figure 1). We used the R package “gcProfileMakeR” to reduce the number of compounds (Pérez-Sanz et al., 2020), based on their frequency, and we applied the random forest classification algorithm. Random forest showed a very low error rate. Only one sample was misclassified, suggesting that differences in floral fragrance among treatments were significant from a mathematical perspective.

The four VOCs found with a higher level of MDA were indole, D-limonene, cinnamyl acetate, and β-myrcene. These compounds were differentially emitted as indole was found only in four out of six conditions, cinnamyl acetate was found in 20-10°C or 20°C while β-myrcene disappeared at constant temperatures. This indicates a possibility to enrich or decrease a single component of a scent profile via changes in growth and/or storage conditions.

Our study showed that the temperature regimens modified scent emission at several levels, including rhythmic emission, emitted quantity, and fragrance composition. Altogether, these modifications resulted in new aroma blends, which can be useful in ornamental and perfume industry. Future perspectives should consider analyzing the internal pool of volatiles as well as the activity of involved enzymes and vaporization mechanisms.

Insights provided reveal that post-harvest conditions affect the scent composition and quantity emitted by narcissi flowers. In addition, floral bouquets composition also changes along the day. These results might be of interest to the perfume and ornamentals industries. Our results show that keeping both a thermoperiod and photoperiod are more appropriate to keep a high-quality scent profile. It is remarkable that the 15°-5°C with 12:12 LD photoperiod resembling the natural winter growing conditions could be considered the best environmental treatment for post-harvest of Narcissus flowers in terms of scent profile.

## Data Availability Statement

The datasets generated for this study are available on request to the corresponding author.

## Author Contributions

MT, VR-H, DÁ, JW, and ME-C conceived and designed the experiments, and corrected the manuscript. DÁ provided the plant material. MT and VR-H sampled and obtained data. MT, VR-H, and DÁ analyzed the data. MT, JW, and ME-C wrote the manuscript. JW and ME-C wrote project and obtained funding. All authors contributed to the article and approved the submitted version.

## Conflict of Interest

DÁ is co-owner of the Las Cabezuelas Sociedad Cooperativa. Samples were provided by the company. The results obtained in the current paper were not influenced by any commercial interest. The remaining authors declare that the research was conducted in the absence of any commercial or financial relationships that could be construed as a potential conflict of interest.
