# Supplementary material for: The Effect of Post-harvest Conditions in Narcissus sp. Cut Flowers Scent Profile
Source: Front Plant Sci. 2021 Jan 7;11:540821. doi: 10.3389/fpls.2020.540821 (PMC7817618; doi:10.3389/fpls.2020.540821)
Supplement: Supplementary file 1 [file Image_1.pdf]

Supplementary Figure S1. Non-constitutive scent profile by frequency. These volatiles were detected in less than 70% of analyzed samples, but with a minimum quality of 80%.

CAS	Volatile	12LD, 15-5 °C	12LD, 20-10 °C	12DD, 15-5 °C	12DD, 20-10 °C	12LD, 5 °C	12LD, 20 °C
000078-79-5	2-Methyl-1,3-butadiene	■	■				
000079-92-5	Camphene	■		■			
000080-26-2	α-Terpineol acetate	■		■			
000080-56-8	2-Pinene				■		
000091-20-3	Naphthalene	■					
000093-58-3	Methyl benzoate	■	■	■	■	■	
000093-89-0	Ethyl benzoate	■		■			
000095-93-2	Durene		■	■		■	
000098-55-5	α-Terpineol	■	■		■		■
000098-86-2	Acetophenone	■	■	■	■	■	
000099-85-4	ɣ-Terpinene	■					
000099-86-5	α-Terpinene	■					
000099-87-6	p-Cymene				■	■	
000100-51-6	Benzyl alcohol	■	■				
000100-52-7	Benzaldehyde		■				
000103-26-4	Methyl cinnamate	■	■				■
000103-54-8	Cinnamyl alcohol, acetate	■	■	■		■	
000104-54-1	Cinnamyl alcohol	■	■				■
000104-55-2	Cinnamaldehyde	■	■		■		
000105-87-3	Geraniol acetate		■				
000106-25-2	Nerol		■				
000120-51-4	Benzyl benzoate		■				■
000120-72-9	Indole					■	
000122-97-4	Benzenepropanol			■	■		■
000123-35-3	β-Myrcene					■	■
000123-92-2	3-Methyl butyl acetate	■	■				■
000124-19-6	Nonanal		■				
000140-29-4	Benzyl nitrile	■		■	■		
000141-12-8	cis-Geranyl acetate						■
000464-17-5	2-Bornene		■				
000470-08-6	β-Fenchyl alcohol	■					
000471-84-1	α-Fenchene				■		
000488-97-1	Cyclofenchene		■		■		
000499-03-6	m-Mentha-1,8-diene						■
000508-32-7	Tricyclene	■					
000514-94-3	α-Pyronene		■		■		
000527-84-4	o-Cymene		■				
000535-77-3	m-Cymene	■	■	■	■	■	■
000586-62-9	α-Terpinolene		■				

000637-69-4	p-Vinylanisole		■				
000673-84-7	Alloocimene			■			■
000874-41-9	Benzene, 1-ethyl-2,4-dimethyl-	■				■	
000934-74-7	Benzene, 1-ethyl-3,5-dimethyl-	■					
001195-32-0	p-Cymenene			■			
001467-36-3	2,3,4-Trimethyl-acetophenone		■				
002040-07-5	2,4,5-Trimethyl-acetophenone		■				
002424-61-5	2-Butenedioic acid (Z)-, monododecyl ester		■				
002870-04-4	2-Ethyl-1,3-dimethylbenzene		■			■	
005989-27-5	D-Limonene				■	■	■
007216-56-0	4-trans,6-cis-Allocimene				■		
007785-26-4	L-α-Pinene			■			
010482-56-1	α-Terpineol			■			
010468-64-1	Benzene, 1-isocyano-2-methyl-	■					
013466-78-9	3-Carene	■	■		■	■	■
017151-09-6	1,2-Bis(trimethylsilyl)benzene	■					
018172-67-3	β-Pinene	■					
021195-59-5	p-Mentha-1,5,8-triene		■		■		■
025155-15-1	Cymene	■	■			■	
025905-14-0	Lavandulol acetate						■
026444-18-8	1-isopropenyl-2-methyl-benzene		■				
055429-29-3	Arsenous acid, tris(trimethylsilyl) ester						■
072237-36-6	(4E)-4-Hexenyl acetate		■				
2000048-79-1	R(+)-Limonene		■				
2000188-30-7	(R)-Lavandulyl acetate						■

Detected (■)

Non-detected
