# Supplementary material for: The Effect of Post-harvest Conditions in Narcissus sp. Cut Flowers Scent Profile
Source: Front Plant Sci. 2021 Jan 7;11:540821. doi: 10.3389/fpls.2020.540821 (PMC7817618; doi:10.3389/fpls.2020.540821)
Supplement: Supplementary file 2 [file Table_1.pdf]

Supplementary Table 1. List of removed siloxanes and their CAS (Chemical Abstracts Services) registry numbers.

CAS	Compound
000141-62-8	Decamethyltetrasiloxane
000540-97-6	Dodecamethylcyclohexasiloxane
000541-02-6	Decamethylcyclopentasiloxane
000541-05-9	Hexamethylcyclotrisiloxane
000556-67-2	Octamethylcyclotetrasiloxane
017928-28-8	Methyltris(trimethylsiloxy)silane
