# Supplementary material for: The Effect of Post-harvest Conditions in Narcissus sp. Cut Flowers Scent Profile
Source: Front Plant Sci. 2021 Jan 7;11:540821. doi: 10.3389/fpls.2020.540821 (PMC7817618; doi:10.3389/fpls.2020.540821)
Supplement: Supplementary file 3 [file Table_2.pdf]

Supplementary Table 2. Principal component analysis (PCA) of narcissi floral aroma at five time points (ZT, *zeitgeber* time). PC: principal component.

ZT		PC1	PC2	PC3	PC4
0	Variance	0.404	0.282	0.190	0.044
	**Factor loading**				
	3,4-Dimethyl 2,4,6-octatriene	-	-	-	-
	Benzenepropanol	-0.335	0.009	0.358	-0.254
	Benzenepropyl acetate	-0.203	-0.440	-0.204	-0.059
	Benzyl acetate	-0.315	-0.301	-0.204	-0.140
	Cinnamyl acetate	-0.312	-0.313	0.011	-0.422
	Eucalyptol	-0.388	0.202	-0.096	0.326
	Indole	-0.018	-0.113	-0.622	-0.260
	Limonene	-0.021	0.388	-0.394	-0.200
	Linalool	-0.402	0.217	-0.093	0.059
	Myrcene	-0.328	0.340	-0.024	0.033
	Ocimene	-0.404	0.037	-0.089	0.423
	Phenethyl acetate	-0.133	0.385	0.271	-0.569
	Pinene	-	-	-	-
	Prenyl acetate	-0.221	-0.317	0.383	0.115
4	Variance	0.358	0.248	0.170	0.107
	**Factor loading**				
	3,4-Dimethyl 2,4,6-octatriene	0.164	0.408	-0.254	0.150
	Benzenepropanol	0.140	-0.067	0.481	0.412
	Benzenepropyl acetate	0.214	-0.042	-0.429	0.320
	Benzyl acetate	0.359	-0.223	-0.187	0.031
	Cinnamyl acetate	0.219	-0.386	-0.176	0.173
	Eucalyptol	0.395	-0.114	0.147	-0.214
	Indole	0.253	-0.150	-0.329	-0.420
	Limonene	0.170	0.418	-0.137	-0.267
	Linalool	0.345	-0.025	0.294	-0.313
	Myrcene	0.242	0.287	0.342	-0.061
	Ocimene	0.431	-0.018	0.117	-0.050
	Phenethyl acetate	0.111	0.396	0.139	0.153
	Pinene	0.119	0.419	-0.263	0.134
	Prenyl acetate	0.304	-0.062	0.005	0.485
16	Variance	0.342	0.238	0.147	0.122
	**Factor loading**				
	3,4-Dimethyl 2,4,6-octatriene	-0.135	0.051	0.621	0.240
	Benzenepropanol	0.115	0.357	0.001	-0.502
	Benzenepropyl acetate	0.208	-0.285	0.126	-0.039
	Benzyl acetate	0.432	-0.136	0.058	0.057
	Cinnamyl acetate	0.367	-0.213	0.041	0.014
	Eucalyptol	0.379	0.275	-0.007	0.099
	Indole	0.368	-0.240	0.036	0.227
	Limonene	-0.053	0.204	-0.265	0.573

	Linalool	0.395	0.163	-0.098	0.195
	Myrcene	-0.006	0.446	-0.013	0.105
	Ocimene	0.277	0.393	0.052	0.116
	Phenethyl acetate	-0.119	0.375	-0.026	0.002
	Pinene	-0.117	0.067	0.622	0.251
	Prenyl acetate	0.247	0.157	0.350	-0.413
22	Variance	0.468	0.275	0.100	0.054
	**Factor loading**				
	3,4-Dimethyl 2,4,6-octatriene	0.172	0.440	0.024	-0.192
	Benzenepropanol	-0.059	0.022	0.756	0.351
	Benzenepropyl acetate	0.364	0.087	-0.075	-0.122
	Benzyl acetate	0.377	-0.071	-0.081	-0.001
	Cinnamyl acetate	0.337	0.160	0.100	-0.096
	Eucalyptol	0.349	-0.160	-0.019	0.212
	Indole	0.258	-0.279	-0.190	-0.053
	Limonene	-0.115	0.186	-0.562	0.587
	Linalool	0.348	-0.032	0.020	0.322
	Myrcene	0.062	0.432	0.151	0.387
	Ocimene	0.357	-0.098	0.003	0.262
	Phenethyl acetate	0.118	0.450	0.029	-0.098
	Pinene	0.104	0.435	-0.098	-0.251
	Prenyl acetate	0.314	-0.200	0.144	-0.170
24	Variance	0.553	0.287	0.077	0.046
	**Factor loading**				
	3,4-Dimethyl 2,4,6-octatriene	-0.349	0.001	0.158	-0.007
	Benzenepropanol	-0.158	-0.424	0.118	0.251
	Benzenepropyl acetate	-0.197	-0.376	0.008	-0.170
	Benzyl acetate	-0.334	0.121	-0.163	0.224
	Cinnamyl acetate	-0.332	-0.113	-0.041	-0.375
	Eucalyptol	-0.355	-0.050	-0.058	-0.059
	Indole	0.168	-0.375	-0.009	0.484
	Limonene	0.146	-0.028	-0.850	-0.278
	Linalool	-0.288	-0.268	-0.174	0.130
	Myrcene	0.093	-0.419	-0.309	0.232
	Ocimene	-0.052	0.420	-0.212	0.553
	Phenethyl acetate	-0.334	0.099	-0.141	0.105
	Pinene	-0.356	-0.021	0.011	-0.021
	Prenyl acetate	-0.290	0.272	-0.127	0.120
